# A phase IV, multi-centre, randomized clinical trial comparing two pertussis-containing vaccines in pregnant women in England and vaccine responses in their infants

**DOI:** 10.1186/s12916-021-02005-5

**Published:** 2021-06-08

**Authors:** Christine Elizabeth Jones, Anna Calvert, Jo Southern, Mary Matheson, Nick Andrews, Asma Khalil, Hannah Cuthbertson, Bassam Hallis, Anna England, Paul T. Heath, Elizabeth Miller

**Affiliations:** 1 Paediatric Infectious Diseases Research Group, St George’s, University of London, London, UK; 2 St George’s University Hospitals NHS Foundation Trust, London, UK; 3 Faculty of Medicine and Institute for Life Sciences, University of Southampton and NIHR Southampton Clinical Research Facility and NIHR Southampton Biomedical Research Centre, University Hospital Southampton NHS Foundation Trust, Clinical and Experimental Sciences, Room LF102, F Level, South Academic Block, Tremona Road, Southampton, SO16 6YD UK; 4 Immunisation and Countermeasures, National Infection Service, Public Health England, London, UK; 5 National Infection Service, Public Health England, Porton, Salisbury, UK; 6 Statistics, Modelling and Economics Department, Public Health England, London, UK

**Keywords:** Maternal vaccination, Immunization, Infant, Immune, Response, Pregnancy, Vaccine

## Abstract

**Background:**

Pertussis vaccines containing three or five pertussis antigens are recommended in pregnancy in many countries, but no studies have compared the effect on infants’ antigen-specific immunoglobulin G (IgG) concentrations. The aim of this study was to compare anti-pertussis IgG responses following primary immunization in infants of mothers vaccinated with TdaP_5_-IPV (low dose diphtheria toxoid, tetanus toxoid, acellular pertussis [five antigens] and inactivated polio) or TdaP_3_-IPV in pregnancy (three pertussis antigens).

**Methods:**

This multi-centre phase IV randomized clinical trial was conducted in a tertiary referral centre and primary care sites in England. Women were randomized to receive TdaP_5_-IPV (*n* = 77) or TdaP_3_-IPV (*n* = 77) at 28–32 gestational weeks. A non-randomized control group of 44 women who had not received a pertussis-containing vaccine in pregnancy and their 47 infants were enrolled post-partum.

**Results:**

Following infant primary immunization, there was no difference in the geometric mean concentrations (GMCs) of anti-pertussis toxin, filamentous haemagglutinin or pertactin IgG between infants born to women vaccinated with TdaP_5_-IPV (*n* = 67) or TdaP_3_-IPV (*n* = 63). However, the GMC of anti-pertussis toxin IgG was lower in infants born to TdaP_5_-IPV- and TdaP_3_-IPV-vaccinated mothers compared to infants born to unvaccinated mothers (*n* = 45) (geometric mean ratio 0.71 [0.56–0.90] and 0.78 [0.61–0.98], respectively); by 13 months of age, this difference was no longer observed.

**Conclusion:**

Blunting of anti-pertussis toxin IgG response following primary immunization occurs in infants born to women vaccinated with TdaP_5_-IPV and TdaP_3_-IPV, with no difference between maternal vaccines. The blunting effect had resolved by 13 months of age. These results may be helpful for countries considering which pertussis-containing vaccine to recommend for use in pregnancy.

**Trial registration:**

ClinicalTrials.gov, NCT02145624, registered 23 May 2014

**Supplementary Information:**

The online version contains supplementary material available at 10.1186/s12916-021-02005-5.

## Background

In 2012, an antenatal pertussis vaccination programme was introduced in the United Kingdom (UK) as an outbreak measure following a significant increase in cases of pertussis and of pertussis-related infant deaths [1]. Two vaccines have been recommended for use in pregnancy in the UK programme—REPEVAX-IPV (2012–2014, 2020) and BOOSTRIX-IPV (2014–current)—with high vaccine effectiveness observed for both vaccines [2]. It is assumed that this reflects the increased levels of maternally derived pertussis-specific antibodies present in the first weeks of life in infants born to vaccinated mothers. However, high levels of maternal antibody can interfere with the infant’s response to primary immunization, a phenomenon known as blunting. This has been shown for five- and three-pertussis antigen component vaccines, but no study has directly compared these vaccines with respect to their impact on infant responses to primary immunization [3–8].

## Methods

The primary objective of this trial was to assess antibody responses to pertussis antigens following primary immunization in infants born to women who were randomized in pregnancy to receive one of two pertussis-containing vaccines and to compare these responses to those in infants born to unvaccinated women. Transplacental transfer and concentrations of pertussis-specific antibodies prior to primary vaccination and 13 months of age were also assessed.

### Study design

In this phase IV, multi-centre, randomized clinical trial, pregnant women were randomized to receive either REPEVAX-IPV or BOOSTRIX-IPV (Additional file 1: Protocol). A contemporaneous non-randomized control group of infants born to women who had not received a pertussis-containing vaccine in pregnancy were also recruited.

Pregnant women, aged 16–45 years at enrolment, receiving antenatal care at St George’s University Hospitals NHS Foundation Trust, or in primary care sites in Gloucestershire and Hertfordshire, were eligible to participate. Exclusion criteria included a bleeding disorder, receipt of a pertussis-containing vaccine in the previous 12 months, receipt of a blood product within the preceding 3 months and any contraindication to vaccination specified in the “Green Book” Immunisation against Infectious Disease [9]. Infants born to women who had not received a pertussis-containing vaccine in the previous 12 months were excluded if there was any contraindication to vaccination specified in the “Green Book” Immunisation against Infectious Disease [9]. Infants were included irrespective of gestational age at birth.

Following written, informed consent, pregnant women were randomized 1:1 to receive either REPEVAX-IPV (2 International Units [IU] diphtheria toxoid [DT], 20 IU tetanus toxoid [TT], 2.5 μg pertussis toxoid, 5 μg filamentous haemagglutinin [FHA], 3 μg pertactin [PRN], 5 μg fimbriae [FIM] types 2 and 3 and inactivated poliovirus [IPV, 40 D-antigen unit Type 1, 8 D-antigen unit Type 2, 32 D-antigen unit Type 3]; TdaP_5_-IPV; Sanofi Pasteur) or BOOSTRIX-IPV (2 IU DT, 20 IU TT, 8 μg pertussis toxoid, 8 μg FHA, 2.5 μg PRN and IPV [40 D-antigen unit Type 1, 8 D-antigen unit Type 2, 32D-antigen unit Type 3]; TdaP_3_-IPV; GlaxoSmithKline (GSK)).

A computerized block randomization list was generated by the study statistician, with sites allocated blocks of sequential numbers (block size 8).

Visits for pregnant women occurred at 28–32 weeks of gestation, up to 7 days post-partum, and at 13 months following delivery. Cord blood or, if not obtained, peripheral blood up to 7 days of age, was collected from infants born to vaccinated mothers. All infants had peripheral blood collected at 2 months (49–84 days of age), 5 months (21–42 days after third primary vaccinations) and 13 months of age (21–42 days after routine booster vaccines). The study was approved by the MHRA, NHS Health Research Authority and City & East Research Ethics Committee (14/LO/0141). The study was registered with ClinicalTrials.gov (NCT02145624) prior to study commencement.

### Intervention

Pregnant women received either TdaP_5_-IPV or TdaP_3_-IPV as a 0.5-ml intramuscular injection into the left upper arm at 28–32 weeks of gestation. Infants received routine vaccines according to the nationally recommended schedule at the time of the study (Table 1).

**Table 1:** The routine immunization schedule recommended in the UK during the study period

Vaccine	Trade name and manufacturer	Recommended age at vaccination (months)
DTaP_3_-IPV-Hib	Infanrix-IPV-Hib, GSK	2, 3, 4
Meningococcal serogroup B	Bexsero, GSK	2, 4, 12
13-valent pneumococcal conjugate	Prevenar13, Pfizer	2, 4, 12
Oral live attenuated rotavirus	Rotarix, GSK	2, 3
Meningococcal serogroup C conjugate	NeisVac-C, GSK	3
Hib-MenC	Menitorix, GSK	12
Measles-mumps-rubella (MMR)	Priorix, Sanofi Pasteur	12

*Abbreviations*: *DTaP*_*3*_*-IPV* diphtheria, tetanus, acellular pertussis (three pertussis antigens, 25 μg each of pertussis toxoid and filamentous haemagglutinin and 8 μg of pertactin), inactivated poliovirus, *Haemophilus influenzae* type B, *IPV* inactivated poliovirus, *Hib Haemophilus influenzae* type b, *MenC* meningococcal serogroup C, *GSK* GlaxoSmithKleine

## Outcomes

The primary outcome was fold difference in anti-pertussis toxin (PT) immunoglobulin G (IgG) geometric mean concentration (GMC) in infants at 5 months of age whose mothers received TdaP_5_-IPV or TdaP_3_-IPV in pregnancy. Secondary outcomes included placental transfer of IgG to pertussis antigens in infants born to vaccinated mothers and GMC in infants born to vaccinated and unvaccinated mothers at 2, 5 and 13 months of age.

### Safety

Women were observed for 20 min post-vaccination for any immediate reaction. Adverse events and serious adverse events (SAEs) were collected for women and infants at each study visit.

### Laboratory assays

Serum IgG to PT, FHA, PRN and FIM 2&3 were quantified using enzyme-linked immunosorbent assays (ELISAs), developed in-house and performed by staff blinded to group allocation. All assays have been validated in accordance with International Conference on Harmonisation guidelines and use the 1st World Health Organization (WHO) International Standard Pertussis Antiserum (human) 06/140 (NIBSC, Item No. 06/140). The lower limit of detection (LLOD) of the assays are 2.128 IU/ml (PT), 0.715 IU/ml (FHA), 0.806 IU/ml (PRN) and 0.636 U/ml (FIM 2&3) with results less than the LLOD, assigned a value half of the LLOD.

### Statistical methods

Sample size calculation was based on the standard deviation of the post-primary vaccination anti-PT IgG GMC of 0.28 IU/ml from a previous study, generated using the same validated PT ELISA [10]. A sample size of 65 per study arm enabled detection of 1.38-fold differences or greater between study arms with 80% power at a 5% significance level. To allow for loss to follow-up, the target sample size was 75–80 in each vaccinated group, with 50 mother-infant pairs in the non-randomized control group.

To increase power, the data for infants born to unvaccinated mothers was supplemented with data from 19 infants from another study conducted at the same sites, at a similar time for which laboratory analysis was performed by the same laboratory using the same assays (NCT01896596). Infants in this study received Infanrix hexa (GSK) instead of Infanrix-IPV-Hib at 2, 3 and 4 months and had been randomized to receive one of three different Men C vaccines at 3 months of age. Blood samples were collected at 5 and 13 months.

A modified intention-to-treat analysis was performed; a per-protocol analysis was not performed as there were fewer than 10% of individuals with data that differed between these populations. Pertussis IgG GMCs and geometric mean fold ratios (GMR) between groups, with 95% confidence intervals (CI), were calculated (Additional materials 2: Statistical Analysis Plan). Normal errors regression on log-transformed data was used to investigate the effect of pre-primary antibody on post-primary GMCs. Two-sided 5% significance was shown when the 95% CI for the GMR or fold effect of pre-primary antibody did not contain unity. The placental transfer ratio (PTR) was calculated as the geometric mean ratio of infant-to-mother-specific IgG at delivery; normal errors regression on logged ratios with adjustment for the interval between vaccination and birth were calculated. The effect of interval from birth to blood test, sex, ethnicity, birth weight and previous maternal pertussis vaccination was also examined by multiple regression when assessing transfer ratios. The proportion of mothers and infants experiencing SAEs was calculated for each group. Missing data were random and excluded from analyses and analyses were performed using Stata version 13.

## Results

**Fig. 1:**
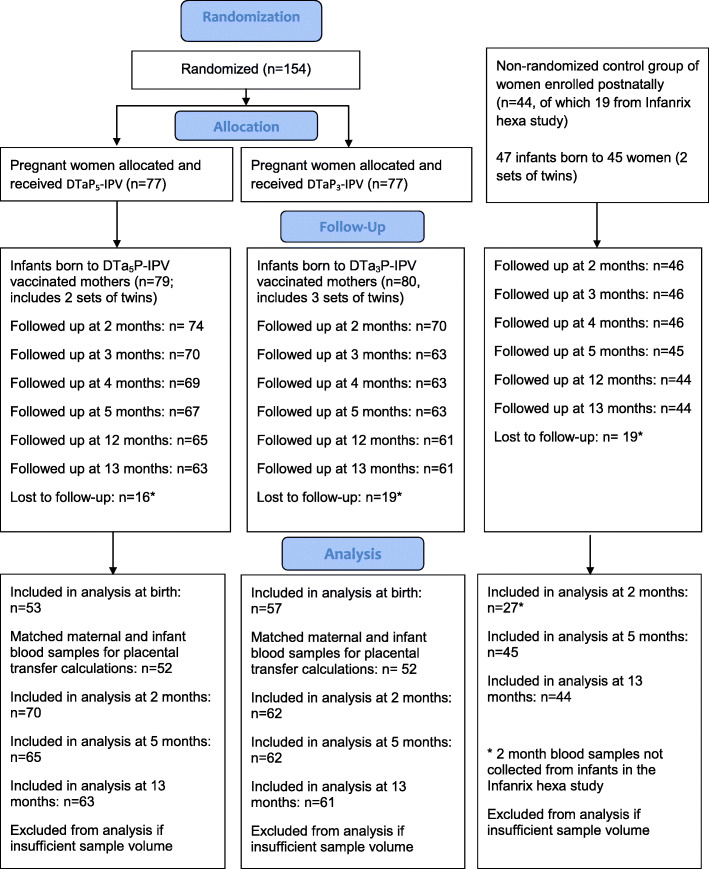
CONSORT flow diagram: randomized groups of women receiving either TdaP_5_-IPV or TdaP_3_-IPV in pregnancy and their infants and a non-randomized group of women not receiving a pertussis-containing vaccine in pregnancy. *Lost to follow-up reasons: Did not return for study visits, moved away from area or vaccines given by primary care provider

Between October 2014 and October 2015, 154 pregnant women were enrolled and randomized to receive either TdaP_5_-IPV (*n* = 77) or TdaP_3_-IPV (*n* = 77); 159 infants were born to these women, and 144 were included in the study (Fig. 1). Twenty-five women who had not received a pertussis-containing vaccine in pregnancy were recruited in the postnatal period, to whom 27 infants were born; data from an additional 19 infants of unvaccinated mothers were included from the Infanrix hexa study.

Demographic and clinical characteristics of participating mother-infant pairs are described in Table 2. There were no baseline demographic differences between groups of women.

**Table 2:** Baseline demographics of participating women and infants

**Characteristic**	**Maternal TdaP**_**3**_**-IPV (*n*** **= 77)**	**Maternal TdaP**_**5**_**-IPV (*n*** **= 77)**	**Unvaccinated mothers (*n*** **= 25)**	**Unvaccinated mothers from Infanrix hexa study (*n*** **= 18)**
Women				
Age, years, median (range)	34 (20–42)	32 (21–44)	31 (20–47)	
Ethnicity, n (%)				
White	63 (82%)	63 (82%)	17 (68%)	16 (89%)
Asian	6 (8%)	7 (9%)	2 (8%)	1 (6%)
Black	3 (4%)	3 (4%)	5 (20%)	0
Chinese	1 (1%)	1 (1%)	0	0
Mixed	3 (4%)	3 (4%)	1 (4%)	1 (6%)
Others	1 (1%)	0	0	0
Site, n (%)				
Gloucestershire, UK	3 (4%)	5 (6%)	0	5 (28%)
Hertfordshire, UK	33 (43%)	28 (36%)	3 (12%)	3 (17%)
South London, UK	41 (53%)	44 (57%)	22 (88%)	10 (56%)
Median gestation at vaccination, week (range)	29 (28–34)	29 (28–33)		
Median interval from antenatal vaccination to delivery, weeks (range)	10 (1–14)	10 (3–14)		
Median interval from delivery to blood sample post-delivery, days (range) [number of samples]	3 (0–10) [51]	2 (0–9) [57]		
	**Maternal TdaP**_**3**_**-IPV (*n*** **= 70)**	**Maternal TdaP**_**5**_**-IPV (*n*** **= 74)**	**Unvaccinated mothers (*n*** **= 27)**	**Unvaccinated mothers from Infanrix hexa study (*n*** **= 19)**
Infants				
Twin, n (%)	6 (9%)	4 (5%)	4 (15%)	2 (11%)
Female, n (%)	33 (42%)	38 (51%)	13 (48%)	10 (52%)
Median gestation at birth, weeks (range)	39 (31–42)	40 (33–42)	38.5 (34–42)	39 (37–42)
Median birth weight, kg (range)	3.39 (1.54–4.69)	3.52 (1.72–4.93)		3.42 (2.74–4.20)
Cord blood collected, n (%)	20 (29%)	21 (28%)		
Infant peripheral blood collected, n (%)	37 (53%)	32 (43%)		
Median interval from birth to 1st infant blood sample, days (range) [number of samples]	3 (0–10) [57]	2 (0–9) [53]		
Median age at pre-vaccination blood sample and vaccine dose 1, days (range) [number of samples]	60 (53–87) [62]	62 (50–80) [70]	64 (54–83) [27]	58 (54–81) [19]
Median age at vaccine dose 2, days (range) [number of samples]	89 (81–113) [63]	91 (81–108) [69]	93 (82–125) [27]	89 (82–121) [19]
Median age at vaccine dose 3, days (range) [number of samples]	120 (108–154) [63]	116.5 (110–143) [68]	123 (110–154) [27]	122 (112–156) [19]
Median interval to blood sample after vaccine dose 3, days (range) [number of samples]	28 (21–47) [62]	28 (21–55) [65]	28 (21–52) [27]	28 (21–40) [18]
Median age at “1 year” vaccination, days (range) [n vaccinated]	372 (351–404) [61]	372 (358–413) [65]	373 (366–394) [27]	377 (354–395) [17]
Median interval from “1 year” vaccination to blood sample, days (range) [number of samples]	28 (21–48) [61]	29 (21–42) [63]	29 (21–52) [27]	31 (21–51) [17]

*Abbreviations*: *TdaP*_*3*_*-IPV* low dose diphtheria, tetanus, acellular pertussis (three pertussis antigens), inactivated poliovirus, *TdaP*_*5*_*-IPV* low dose diphtheria, tetanus, acellular pertussis (five pertussis antigens), inactivated poliovirus, *UK* United Kingdom

The following cells are empty as data was not collected from the women recruited in the postnatal period who had not received a pertussis-containing vaccine in pregnancy: maternal age (Infanrix hexa group only), gestation at vaccination, median interval from antenatal vaccination to delivery, median interval from delivery to blood sample post-delivery, birth weight, number of cord blood samples or peripheral blood at birth collected, and median interval from birth to 1st infant blood sample

### Placental transfer (Table 3)

The PTR of IgG was greater than 1 for all pertussis antigens, with no difference observed according to maternal vaccine received. There was a strong positive correlation between infant and maternal GMCs of IgG for PT, FHA, FIM 2&3 and PRN. The placental transfer ratio increased with increasing time from vaccination to birth of the infant.

**Table 3:** Pertussis-antigen-specific IgG GMC in mothers at delivery and placental transfer from TdaP_3_-IPV- and TdaP_5_-IPV-vaccinated mothers to their infants

	Maternal TdaP3-IPV				Maternal TdaP5-IPV				TdaP5-IPV:TdaP3-IPV	TdaP_5_-IPV and TdaP_3_-IPV combined	
Antigen	N	Maternal GMC (95% CI) at delivery of infant	N	Geometric mean placental transfer ratio (95% CI)	N	Maternal GMC (95% CI) at delivery of infant	N	Geometric mean placental transfer ratio (95% CI)	Adjusted^a^ ratio of placental transfer ratios (95% CI)	Correlation coefficient^b^ for infant vs maternal GMC	Fold change in GMC per week, from maternal vaccination to birth^a^
PT	51	37.46 (28.88–48.57)	51	1.37 (1.24–1.52)	57	23.77 (18.1–31.21)	52	1.60 (1.47–1.73)	1.11 (0.99–1.24)	0.95	1.08 (1.05–1.10)
FHA	51	163.96 (128.67–208.92)	51	1.47 (1.34–1.62)	57	68.86 (56.04–84.63)	52	1.51 (1.35–1.68)	0.95 (0.85–1.06)	0.93	1.10 (1.08–1.13)
FIM	51	22.97 (15.19–34.72)	51	1.41 (1.29–1.54)	57	195.71 (127.17–301.19)	52	1.58 (1.43–1.74)	1.07 (0.95–1.21)	0.99	1.06 (1.04–1.09)
PRN	51	186.06 (118.55–292.02)	50	1.37 (1.21–1.55)	57	181.38 (125.15–262.86)	50	1.40 (1.25–1.57)	0.95 (0.83–1.09)	0.96	1.11 (1.08–1.14)

*Abbreviations*: *TdaP*_*3*_*-IPV* low dose diphtheria, tetanus, acellular pertussis (three pertussis antigens), inactivated poliovirus, *CI* confidence interval, *TdaP*_*5*_*-IPV* low dose diphtheria, tetanus, acellular pertussis (five pertussis antigens), inactivated poliovirus, *PT* pertussis toxin, *FHA* filamentous haemagglutinin, *FIM* fimbriae types 2 and 3, *PRN* pertactin, *GMC* geometric mean concentration

^a^Adjusted for interval from maternal vaccination to birth of infant; ^b^Pearson correlation coefficient

Multivariable analysis was performed to identify the factors associated with PTR. Time from vaccination to birth was significantly associated with PTR, with a fold change of 1.08 (95% CI 1.05–1.10), 1.10 (1.08–1.13), 1.06 (1.04–1.09) and 1.11 (1.08–1.14) for PT, FHA, FIM 2&3 and PRN respectively per week. If a cord sample was not collected, the time at which the infant peripheral blood sample was obtained was significantly associated with PTR with a fold change of 0.97 (0.96–0.99), 0.97 (0.95–0.99), 0.98 (0.96–1.00) and 0.99 (0.96–1.01) for PT, FHA, FIM and PRN respectively per day from birth to blood sampling. Other factors assessed were not associated with the PTR.

### Infant antibody concentrations at 2 months of age (Table 4)

Prior to primary vaccination, infants born to mothers vaccinated with TdaP_5_-IPV had lower GMC of anti-PT and FHA IgG compared to infants born to TdaP_3_-IPV-vaccinated mothers (GMR 0.64 [95% CI 0.43–0.94] and 0.48 [0.35–0.65] respectively). Infants born to mothers vaccinated with TdaP_5_-IPV had higher GMCs of anti-FIM IgG (GMR 8.71 [5.2–14.58]). Anti-PRN IgG were similar for infants born to TdaP_5_-IPV- and TdaP_3_-IPV-vaccinated mothers.

**Table 4:** Pertussis-antigen-specific IgG GMC in infants at birth, 2, 5 and 13 months of age

		Maternal TdaP_3_-IPV		Maternal TdaP_5_-IPV		Unvaccinated mothers		TdaP_5_-IPV:DTaP_3_-IPV	TdaP_3_-IPV:none	TdaP_5_-IPV:none
Antigen	Time point	N	GMC (95% CI)	N	GMC (95% CI)	N	GMC (95% CI)	GMR (95% CI)	GMR (95% CI)	GMR (95% CI)
PT	Birth	57	50.06 (37.55–66.75)^a^	53	35.84 (26.73–48.05)			0.72 (0.48–1.07)		
	2 months	61	16.82 (12.64–22.37)	70	10.7 (8.07–14.18)	27	2.64 (1.77–3.94)	0.64 (0.43–0.94)	6.36 (3.82–10.61)	4.05 (2.45–6.68)
	5 months	62	30.72 (26.32–35.84)	65	33.55 (28.89–38.96)	45	43.2 (35.5–52.58)	1.09 (0.88–1.35)	0.71 (0.56–0.9)	0.78 (0.61–0.98)
	13 months	61	5.07 (3.95–6.51)	63	6.05 (4.56–8.01)	44	6.56 (4.79–9.00)	1.19 (0.82–1.73)	0.77 (0.51–1.16)	0.92 (0.62–1.38)
	5:2 months	60	1.89 (1.33–2.69)	65	3.25 (2.29–4.62)	27	14.1 (8.67–22.93)	1.72 (1.05–2.81)	0.13 (0.07–0.25)	0.23 (0.12–0.43)
FHA	Birth	57	245.79 (190.72–316.76)	53	97.44 (78.44–121.03)			0.4 (0.29–0.55)		
	2 months	61	66.37 (51.95–84.79)	70	31.74 (26.05–38.68)	27	5.97 (4.02–8.87)	0.48 (0.35–0.65)	11.12 (7.36–16.79)	5.32 (3.55–7.96)
	5 months	62	54.52 (47.75–62.25)	65	50.67 (43.92–58.45)	45	67.31 (55.02–82.34)	0.93 (0.77–1.13)	0.81 (0.65–1.01)	0.75 (0.6–0.94)
	13 months	61	14.01 (11.25–17.43)	63	18.07 (13.81–23.64)	44	22.2 (17.15–28.72)	1.29 (0.92–1.81)	0.63 (0.44–0.91)	0.81 (0.57–1.17)
	5:2 months	60	0.83 (0.65–1.05)	65	1.67 (1.3–2.15)	27	10.37 (6.31–17.04)	2.02 (1.43–2.84)	0.08 (0.05–0.13)	0.16 (0.1–0.26)
PRN	Birth	56	250.13 (160.52–389.77)	52	230.53 (151.1–351.7)			0.92 (0.51–1.68)		
	2 months	62	62.51 (39.17–99.75)	70	83.02 (59.29–116.25)	27	2.63 (1.44–4.82)	1.33 (0.76–2.32)	23.73 (11.46–49.15)	31.52 (15.41–64.46)
	5 months	62	65.88 (55.66–77.96)	65	53.32 (42.59–66.76)	44	73.85 (54.41–100.22)	0.81 (0.61–1.08)	0.89 (0.64–1.24)	0.72 (0.52–1)
	13 months	60	10.47 (8.18–13.42)	63	7.04 (5.33–9.30)	40	11.24 (8.4–15.04)	0.67 (0.47–0.97)	0.93 (0.62–1.39)	0.63 (0.42–0.93)
	5:2 months	61	1.06 (0.64–1.75)	65	0.65 (0.45–0.93)	27	26.54 (11.81–59.62)	0.62 (0.34–1.12)	0.04 (0.02–0.09)	0.02 (0.01–0.05)
FIM	Birth	56	34.09 (22.77 –51.04)	53	282.36 (177.32–449.62)			8.28 (4.55–15.09)		
	2 months	61	9.82 (6.71–14.37)	70	85.5 (59.59–122.67)	27	5.2 (2.94––9.21)	8.71 (5.2 –14.58)	1.89 (0.96–3.71)	16.43 (8.47–31.88)
	5 months	62	3.64 (2.77–4.79)	65	18.47 (13.58–25.12)	45	2.32 (1.73–3.12)	5.07 (3.38–7.61)	1.57 (1.02–2.41)	7.95 (5.19–12.16)
	13 months	61	1.54 (1.26–1.87)	63	1.49 (1.24–1.79)	44	1.21 (0.92–1.60)	0.97 (0.75–1.26)	1.27 (0.93–1.72)	1.23 (0.91–1.67)
	5:2 months	60	0.38 (0.3–0.48)	65	0.22 (0.19–0.26)	27	0.49 (0.33–0.71)	0.58 (0.44–0.76)	0.79 (0.55–1.14)	0.46 (0.32–0.66)

*Abbreviations*: *DdaP*_*3*_*-IPV* low dose diphtheria, tetanus, acellular pertussis (three pertussis antigens), inactivated poliovirus, *TdaP*_*5*_*-IPV* low dose diphtheria, tetanus, acellular pertussis (five pertussis antigens), inactivated poliovirus, *GMC* geometric mean concentration, *CI* confidence interval, *GMR* geometric mean ratio, *PT* pertussis toxin, *FHA* filamentous haemagglutinin, *FIM* fimbriae types 2 and 3, *PRN* pertactin

^a^The GMC of anti-PT IgG was lower than the limit of detection for one infant at birth

Blood samples were not collected at birth from infants born to women not receiving a pertussis-containing vaccine in pregnancy as these women and infants were only recruited in the postnatal period; therefore, corresponding cells are empty

Compared to infants in the control group, infants born to vaccinated women had higher GMCs of anti-PT, FHA, FIM 2&3 and PRN IgG (GMR 4.05 [2.45–6.68], 5.32 [3.55–7.96], 16.43 [8.47–31.88] and 31.52 [15.41–64.46] respectively for the DTaP_5_-IPV group; GMR 6.36 [3.82–10.61], 11.12 [7.36–16.79], 1.89 [0.96–3.71] and 23.73 [11.46–49.15] respectively for the TdaP_3_-IPV group).

### Infant antibody concentrations at 5 months of age (Table 4)

Following infant primary vaccination at 2, 3 and 4 months, infants born to women vaccinated with TdaP_5_-IPV had similar GMCs of anti-PT, FHA and PRN as infants born to women vaccinated with TdaP_3_-IPV, but higher anti-FIM 2&3 IgG concentrations.

The GMC of anti-PT IgG in infants born to TdaP_5_-IPV- or TdaP_3_-IPV-vaccinated mothers was lower than that in infants born to unvaccinated mothers (GMR 0.71 [0.56–0.90] and 0.78 [0.61–0.98], respectively). For anti-FHA IgG, this effect was only seen in infants born to TdaP_5_-IPV-vaccinated mothers (GMR 0.75 [0.6–0.94]).

GMCs of anti-PT IgG increased from pre- to post-primary vaccination for all infants (TdaP_5_-IPV group: fold change 1.89 [1.33–2.69]; TdaP_3_-IPV group 3.25 [2.29–4.62]; unvaccinated group 14.1 [8.67–22.93]). However, there was no significant difference in the fold change in IgG to any pertussis antigens from pre- to post-primary vaccination in infants born to mothers receiving TdaP_5_-IPV compared to those infants receiving TdaP_3_-IPV.

### Infant antibody concentrations at 13 months of age (Table 4)

At 13 months of age, GMCs of anti-PT, FHA and FIM 2&3 were similar in those infants born to TdaP_3_-IPV- and TdaP_5_-IPV-vaccinated mothers, whereas GMCs of anti-PRN IgG was lower in infants born to TdaP_5_-IPV-vaccinated mothers compared to infants born to DTaP_3_-IPV-vaccinated mothers (GMR 0.67 [0.47–0.97]). Compared to infants whose mothers received neither vaccine in pregnancy, infants born to TdaP_3_-IPV-vaccinated mothers had lower GMCs of anti-FHA (GMR 0.63 0.44–0.91). For anti-PRN IgG, infants born to TdaP_5_-IPV-vaccinated mothers had lower concentrations compared to unvaccinated infants (GMR 0.63 [0.42–0.93]).

### Effect of GMC of pertussis-specific IgG at 2 months of age on GMC post-primary vaccination and at 13 months of age

A higher GMC of anti-PT IgG at 2 months of age was associated with a lower GMC of anti-PT IgG at 5 months of age; the fold change of the post-primary GMC of anti-PT IgG was 0.92 (0.87–0.98) per twofold change in the pre-primary GMC of anti-PT IgG. This effect was not observed at 13 months of age. A similar effect was seen for anti-FHA IgG at 13 months of age (fold change in anti-FHA IgG GMC at 13 months of age 0.87 [95% CI 0.77–0.99] per twofold change in pre-primary GMC), but not at 5 months of age (fold change in post-primary anti-FHA IgG GMC 1.07 [95% CI 0.99–1.14] per 2-fold change in pre-primary GMC). Conversely, GMCs of anti-FIM 2&3 IgG were higher post-primary vaccination with lower concentrations pre-vaccination (fold change in anti-FIM IgG GMC at 5 months of age 1.61 [95% CI 1.55–1.68] per twofold change in pre-primary GMC).

### Safety

Overall, there were 5 maternal SAEs: 2 in women in the TdaP_5_-IPV group, 2 in TdaP_3_-IPV and one in an unvaccinated mother. There were 10 SAEs in infants born to women in the TdaP_5_-IPV group, 4 in infants born to women in the TdaP_3_-IPV group and 3 in infants in the control group. No SAEs were assessed as related to maternal or infant vaccination.

## Discussion

Pertussis vaccination in pregnancy is an effective and safe strategy for the prevention of pertussis in early infancy [2, 4, 11–27]. However, blunting of the infant’s response to primary vaccination may occur in the context of high concentrations of maternally derived antibody. Differences in the pertussis antigen content of vaccines recommended in pregnancy might result in differential blunting of the infant’s response to primary immunization and therefore in the persistence of antibody, and of protection against pertussis, through early childhood. This is particularly important in countries, such as the UK and many low- and middle-income countries, where a pertussis-booster dose is not given until pre-school age.

To our knowledge, this is the first randomized clinical trial of different pertussis-containing vaccines in pregnancy. Other studies have examined only TdaP_5_ (Adacel, Sanofi Pasteur) [4, 5, 7, 8, 28], TdaP_5_-IPV (REPEVAX, Sanofi Pasteur) [3, 29] or TdaP_3_ (BOOSTRIX, GSK) [6, 30, 31] and found differing impacts on infant responses to primary and booster vaccinations, but no study has compared these vaccines directly. This is an important consideration for countries which recommend pertussis vaccination in pregnancy or that are considering implementing such a programme.

Passive infant immunity to pertussis is contingent on efficient transplacental passage of antibody; it is reassuring therefore that there is no difference in the PTR in women vaccinated with either vaccine. As shown in other studies, the newborn infant concentration of antibody in this study was greater than the maternal concentration, with a PTR of greater than one for all antigens [4, 6–8, 29, 32]. Multivariate analysis showed that time from third-trimester vaccination to delivery was positively associated with PTR. This may be because of the effects of cumulative exposure to the maternal antibody; however, placental transfer also becomes more efficient with advancing gestational age [33]. The narrow time window of vaccination and the very small number of preterm deliveries made it hard to separate the influence of these factors.

At 2 months of age, significantly higher concentrations of pertussis-specific antibody were found in infants born to vaccinated women, compared to infants born to unvaccinated women, consistent with the high maternal vaccine efficacy observed in the UK, Australia and the USA [2, 12, 13, 34]. Infants of TdaP_3_-IPV-vaccinated mothers had higher concentrations of anti-PT and FHA IgG compared to infants born to TdaP_5_-IPV-vaccinated mothers, consistent with the increased quantity of pertussis toxoid and FHA in TdaP_3_-IPV compared to TdaP_5_-IPV. Only TdaP_5_-IPV contains FIM2&3 antigens; therefore, infants born to TdaP_5_-IPV-vaccinated mothers had higher concentrations of anti-FIM IgG pre-primary vaccination. There was no difference in anti-PRN IgG GMC, reflecting the similar amount of PRN antigen in both vaccines. Although there is no correlate of protection for pertussis disease, higher levels of pertussis-specific antibodies are associated with protection from disease, in particular anti-PT and PRN IgG [35–38].

Whilst a higher concentration of pertussis-specific antibody is likely to correlate with clinical protection early in infancy, it might also attenuate infant vaccine responses. Reassuringly, we found no significant difference in the concentration of anti-PT, FHA or PRN IgG post-primary vaccination in infants born to TdaP_5_-IPV compared to infants born to TdaP_3_-IPV-vaccinated women, suggesting that either vaccine may be employed within a national antenatal programme, with no difference in blunting seen. When compared to infants born to unvaccinated women, blunting was observed for anti-PT IgG responses, although this effect was not sustained at the end of the first year of life. A modest reduction in anti-FHA IgG was also seen in infants born to TdaP_5_-IPV mothers compared to infants born to unvaccinated mothers, but this was not observed at 13 months of age. We could not assess whether high concentrations of maternally derived anti-FIM IgG would have blunted responses to primary infant immunization with a five-component acellular pertussis (aP) vaccine, since infants in this study did not receive a FIM-containing aP vaccine. However, an earlier non-randomized study did suggest that blunting of the anti-FIM 2&3 response can also occur [3].

Whilst the blunting effect observed was statistically significant, it is important to note that the absolute difference in GMCs was small. It is particularly reassuring that UK epidemiological data did not show an excess of pertussis cases in infants born to vaccinated compared to unvaccinated women following infant primary vaccination [2]. Some studies have also shown blunting post-primary vaccination [5, 6, 8, 31]; however, others have not shown a significant blunting effect [4, 5, 28, 29]. In countries where a booster dose is given in the second year of life, some studies have shown persisting evidence of blunting [8, 30, 31], whilst others have not shown any difference post booster [4, 5, 28]. The latter were more limited in size or had not demonstrated significant blunting post-primary vaccination. Factors which may influence blunting include maternal vaccine received, timing of infant vaccination and the pertussis antigen component of infant vaccination, both in terms of the number and amount of antigen.

### Strengths and limitations

This randomized clinical trial is the first to compare the effect of vaccination with either TdaP_5_-IPV or TdaP_3_-IPV in pregnancy on transplacental transfer of antibody and the serological response to infant primary immunization and the concentration of vaccine-specific antibody at 13 months of age.

We did not include a randomized control group as this would have been unethical in the context of national recommendations for antenatal pertussis vaccination. We recruited the control group of women and infants in the postnatal period to ensure that women were not discouraged from receiving a pertussis-containing vaccine in pregnancy. We had difficulty recruiting to the control group; therefore, included data from infants included in another study, carried out at the same sites using similar protocols and with samples analysed using identical methods in the same laboratory.

The combination vaccine used in the maternal pertussis vaccination programme in the UK contains IPV; to our knowledge, other countries do not use a combination vaccine that includes IPV. Despite this, we do not consider that this would have significantly influenced our results. The UK primary immunization schedule is administered at 2, 3 and 4 months of age; other countries employ different primary immunization schedules; therefore, this might impact on the generalizability of the results.

The study commenced towards the beginning of the antenatal pertussis programme and most women received a pertussis-containing vaccine for the first time in the current pregnancy; further studies should examine the effects of repeat doses of vaccine in subsequent pregnancies.

## Conclusion

We provide robust evidence to suggest that either TdaP_5_-IPV or TdaP_3_-IPV vaccines may be used in pregnancy, with no differential effect on the protection afforded against pertussis by infant primary immunization with an aP_3_ vaccine or that sustained into the second year of life. This data may provide reassurance that either TdaP_5_-IPV or TdaP_3_-IPV may be used within national pertussis vaccination programmes in pregnancy.

## Supplementary Information

**Additional file 1.** Protocol. Full trial protocol.

**Additional file 2.** Statistical analysis plan. Full statistical analysis plan.

## Data Availability

The datasets analysed during the current study may be available from the corresponding author on reasonable request.

## Abbreviations

aP: Acellular pertussis
DT: Diphtheria toxoid
ELISA: Enzyme-linked immunosorbent assay
FHA: Filamentous haemagglutinin
FIM: Fimbriae
GMC: Geometric mean concentration
GMR: Geometric mean ratio
GSK: GlaxoSmithKline
Hib: *Haemophilus influenza*e type b
IgG: Immunoglobulin G
iMAP2: Immunising Mums Against Pertussis
IPV: Inactivated poliovirus
IU: International Units
LLOD: Lower limit of detection
Men C: Meningococcal serogroup C
PRN: Pertactin
PT: Pertussis toxin
PTR: Placental transfer ratio
SAE: Serious adverse event
TdaP_3_-IPV: Low dose diphtheria toxoid, tetanus toxoid, acellular pertussis [three antigens] and inactivated polio
TdaP_5_-IPV: Low dose diphtheria toxoid, tetanus toxoid, acellular pertussis [five antigens] and inactivated polio
TT: Tetanus toxoid
UK: United Kingdom
WHO: World Health Organization
